# Towards the Estimation of Body Weight in Sheep Using Metaheuristic Algorithms from Biometric Parameters in Microsystems

**DOI:** 10.3390/mi13081325

**Published:** 2022-08-16

**Authors:** Enrique Camacho-Pérez, Alfonso Juventino Chay-Canul, Juan Manuel Garcia-Guendulain, Omar Rodríguez-Abreo

**Affiliations:** 1 Tecnológico Nacional de México/Instituto Tecnológico Superior Progreso, Progreso 97320, Mexico; 2 Red de Investigación OAC Optimización, Automatización y Control, El Marques 76240, Mexico; 3 División Académica de Ciencias Agropecuarias, Universidad Juárez Autónoma de Tabasco, km 25, Carretera Villahermosa-Teapa, R/A La Huasteca, Colonia Centro Tabasco 86280, Mexico; 4 Industrial Technologies Division, Universidad Politécnica de Querétaro, El Marques 76240, Mexico

**Keywords:** artificial intelligent, embedded microsystems, genetic algorithm, cuckoo search algorithm, weight estimation, computer vision, mathematical model

## Abstract

The Body Weight (BW) of sheep is an important indicator for producers. Genetic management, nutrition, and health activities can benefit from weight monitoring. This article presents a polynomial model with an adjustable degree for estimating the weight of sheep from the biometric parameters of the animal. Computer vision tools were used to measure these parameters, obtaining a margin of error of less than 5%. A polynomial model is proposed after the parameters were obtained, where a coefficient and an unknown exponent go with each biometric variable. Two metaheuristic algorithms determine the values of these constants. The first is the most extended algorithm, the Genetic Algorithm (GA). Subsequently, the Cuckoo Search Algorithm (CSA) has a similar performance to the GA, which indicates that the value obtained by the GA is not a local optimum due to the poor parameter selection in the GA. The results show a Root-Mean-Squared Error (RMSE) of 7.68% for the GA and an RMSE of 7.55% for the CSA, proving the feasibility of the mathematical model for estimating the weight from biometric parameters. The proposed mathematical model, as well as the estimation of the biometric parameters can be easily adapted to an embedded microsystem.

## 1. Introduction

Livestock Body Weight (BW) is often used as a tool in feeding strategies [1,2], to estimate the intestinal microbiota [3], to assess growth [4], to assess the effects of heat stress [5], and to evaluate genetic selection [6]. In general, it has been an important parameter for both the research on and management of commercial farms around the world.

There are two principal methodologies to estimate the BW: the first is through direct measurements via scales, and the second is based on indirect measures of different parts of the body [7]. For indirect weight estimation, several methodologies have been implemented, such as those that use mathematical regression models to estimate the BW through morphological measurements [8,9]. Recently, these measurements have been improved with the application of technology, from digital images [10,11,12,13,14,15,16], stereo vision [17], Light Detection And Ranging (LiDAR) sensors [18], 3D images [19,20], to depth sensors [21,22,23]. Among the latter mentioned innovations, the Kinect^®^ arises due to its low cost and easy implementation. It is used to estimate the weight, as well as for other livestock applications, such as measuring live cattle body parameters [24], the estimation of the muscle scores of live pigs [25], and sheep behavior recognition [26], among other studies.

The advancement of new technologies, studies, and processes in livestock has been complemented by Computer Vision (CV), which consists of a set of techniques used to analyze visual information, including its acquisition, transmission, processing, and understanding [27]. Some applications of this technology include the recognition of the behavior of pigs [28], cattle monitoring [29], automatic heat detection in cows [30], segmentation of body parts [31], and a wide variety of studies related to BW estimation, which are summarized in [32]. In addition to CV, new prediction models have also been implemented, especially those based on deep learning [33,34,35,36], which improves their performance when more information is provided. However, other methodologies, such as Metaheuristic Algorithms (MAs), do not require much data, which can be used to solve optimization or forecast problems. So far, there are few applications of metaheuristic algorithms to livestock; the few reported applications are for the optimization of resources [37,38] and logistics [39], but there are no reports regarding their direct application for the estimation of biometric parameters such as the ones presented here.

MAs are essentially high-level strategies that employ some specific tactics for exploring the search space to find a near-optimal solution(s) [40]. MAs have the most significant advantage over other methods because of their flexibility, which allows them to address a wide range of scientific problems in many areas, especially in engineering. Their flexibility is because they do not depend on the mathematical model of the studied system. Their simplicity and easy implementation allow them to solve most optimization and search problems [41]. The use of MAs as parameter estimators has been tested in various engineering applications, for example, in [42], the parameters of a motor were estimated from the angular speed and the current it consumed. Another example is the investigation in [43], in which the Rough-Enhanced-Bayes (REBMIX) mixture estimation algorithm was used to estimate the parameters of a mixture model.

Therefore, using two MAs is proposed as a novel method to estimate the BW: Genetic Algorithms (GAs) and the Cuckoo Search Algorithms (CSAs). The GA is commonly used to generate optimization and search problems, often employing the processes of natural evolution, such as inheritance, mutation, selection, and crossover. The GA has been shown to perform well in mixed (continuous and discrete) combinatorial problems [44]. The CSA proposed by Yang and Deb [45] is based on the Lévy flight behavior (or random walk model) and the parasitic behavior of broods. Cuckoos reproduce by laying their eggs in the nests of other birds for them to raise and feed their young. The cuckoos with the highest breeding success are the ones that lay eggs that are the most similar to their host’s, as an egg that is different will be detected and the host will either drop the egg or leave the nest. If a cuckoo’s egg in a host’s nest is considered a possible solution, the worst solutions will be abandoned, and new solutions will be sought. The new solutions will be searched through a Lévy flight, a search model of many animals and insects. In this type of flight, the animal searches in a straight path, then suddenly changes its direction, leading to an irregular scale-free search pattern. This type of flight benefits the search for new solutions as new areas in the search space are explored. These characteristics allow the CSA to be a highly efficient option for solving optimization problems in continuous functions [46].

There is a lack of published information on the use of metaheuristic algorithms to estimate important biometric parameters of livestock. Most of the works deal with issues of resource optimization. For example, in [47], the study attempted to posit a suitable strategy for optimal production with maximum resilience and sustainability in industrial dairy farms. Another type of application was given in [48], where a metaheuristic approach was presented for selecting optimal clusters in wireless body area networks to achieve an energy-efficient routing protocol for livestock health and behavior monitoring. In [37], two metaheuristic algorithms, MOPSO and MICA, were used to balance the broiler production process’s cost, time, and quality.

Currently, in the livestock field, there is interest in monitoring parameters through embedded systems, for example, in [49], the implementation of an electronic system specifically developed for real-time monitoring of feeding patterns in dairy cows was shown. In [50], an embedded system was presented that could track individual cows using ultra-wideband technology; in addition, social interactions between individuals around the feeding area were analyzed with a computer vision module. This work aims to develop a model based on biometric parameters to estimate the BW, using the GA and the CSA through a polynomial model with a variable degree adjusted by MAs; these biometric parameters can be acquired through a CV system that is suitable for implementation on embedded systems. Therefore, given many variables and taking advantage of the available information, a polynomial model is proposed, which has shown promising results in other areas [51,52].

Two algorithms are used to verify that the efficiency of the GA is not limited to a local optimum. Unlike the works described above, the proposal of this work uses the evolutionary computation branch to estimate the BW. This technique has an advantage over intelligent techniques based on Artificial Neural Networks (ANNs), which present a polynomial model and not a black box one, as in an ANN. The results show a Root-Mean-Squared Error (RMSE) of 7.68% for the GA and 7.55% for the CSA.

## 2. Materials and Methods

**Figure 1:**
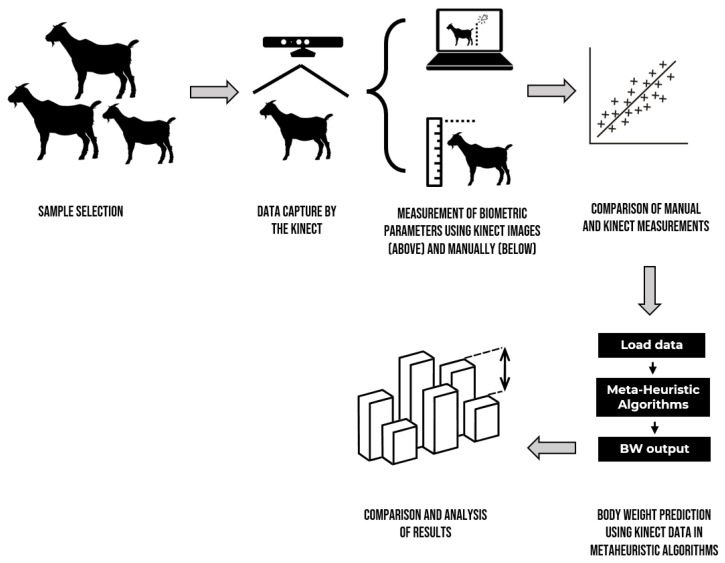
Workflow of the main stages carried out in this study.

In this section, each of the stages for the construction of the mathematical model is developed in detail: from the handling of animals to the application of the metaheuristic algorithms. Figure 1 briefly shows the workflow of this study.

### 2.1. Animals, Diets, and Handling

In this investigation, were worked with Pelibuey sheep, which is a type of sheep that generally is not raised for wool. The animals were handled according to the Regulations for Ethical Animal Experimentation of the División Académica de Ciencias Agropecuarias of the Universidad Juárez Autónoma de Tabasco (ID project PFI: UJAT-DACA-2015-IA-02). The experiment was performed at the Southeastern Center for Ovine Integration (17°$78^{\prime }$ N, 92°$96^{\prime }$ W; 10masl). The data were collected from 56 non-pregnant and non-lactating Pelibuey ewes aged from four to 10 months with a mean BW of 30.31±7.83kg. The animals for the experiment were placed in raised slatted-floor cages with a feeding group in a feedlot system. The experimental diet was a total mixed ration (80:20 concentrate to forage ratio) comprising ground maize, soybean meal, star grass hay, vitamins, and mineral premix and had a crude protein level of 16% Dry Matter (DM) [53].

### 2.2. Image Acquisition

The Python programming language [54] and the Numpy [55], OpenCV [56], and Pandas [57] libraries were used to process and manipulate the images and the data obtained manually. The procedure followed is described next. First, an image acquisition program was developed to acquire data from a Kinect^®^ sensor (Version 1) and implemented on a computer for data collection. For data acquisition, it should be considered that light is the sensed variable. Thus, sudden brightness changes should be avoided to obtain an adequate contrast between the animal and the background. Although the processing has a tolerance for changes, the measurements were made under a roof to prevent excess light. On the other hand, the sensor was ideally placed at 1.5 m. The camera calibration worked as long as the animal stayed between 1.2 and 1.8 m.

**Figure 2:**
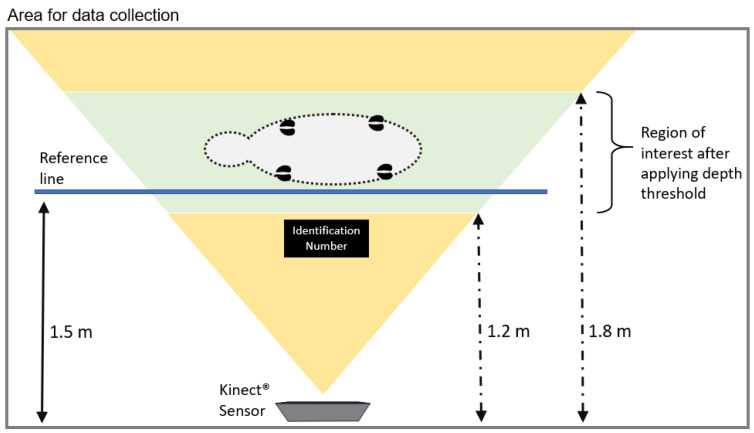
Configuration of the area for data collection.

The Kinect^®^ sensor was placed on the side of the animal, with a separation distance of approximately 1.5m, allowing the entire body length’s registration. A reference line was added; a label with the identification number was placed on each animal, which helped identify them in the subsequent analysis (Figure 2).

**Figure 3:**
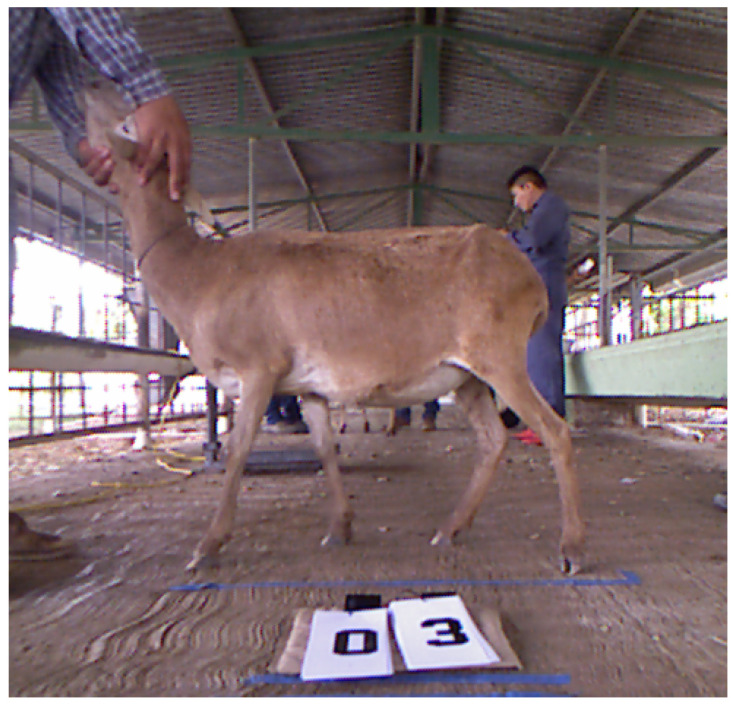
RGB image from the Kinect^®^ sensor.

**Figure 4:**
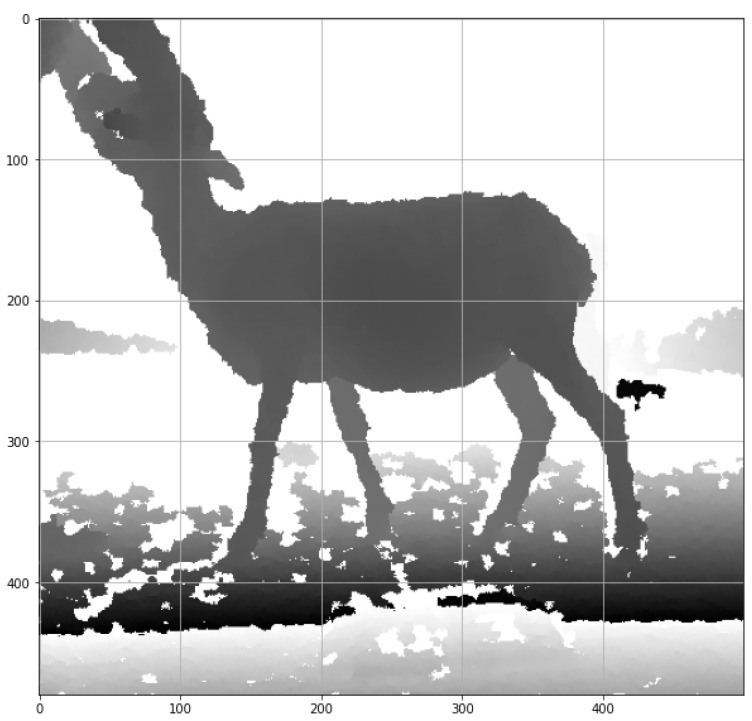
Depth image from the Kinect^®^ sensor.

With the Kinect^®^ sensor, two images were obtained, a color image with a resolution of 640 × 480 pixels (Figure 3) and a depth image of 320 × 240 pixels (Figure 4). Each image was taken and recorded every second. Next, the color image was saved in a Portable Network Graphics (PNG) file and the depth file in the Comma-Separated Values (CSV) format. Finally, this last file was processed through the OpenCV and Numpy libraries of the Python programming language.

**Figure 5:**
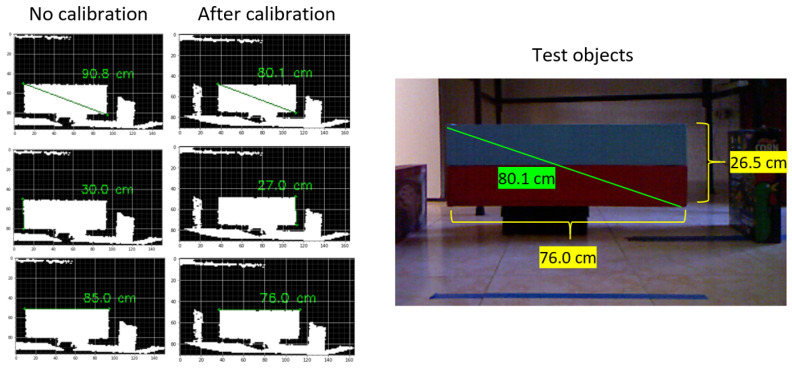
Calibration tests.

It was necessary to calibrate the depth images to obtain the actual measurements in the three spatial coordinates, which followed the procedure suggested in [58]. The calibration was tested on objects with regular shapes and some biometric measurements of sheep to ensure that the measurements were correct (see Figure 5).

**Figure 6:**
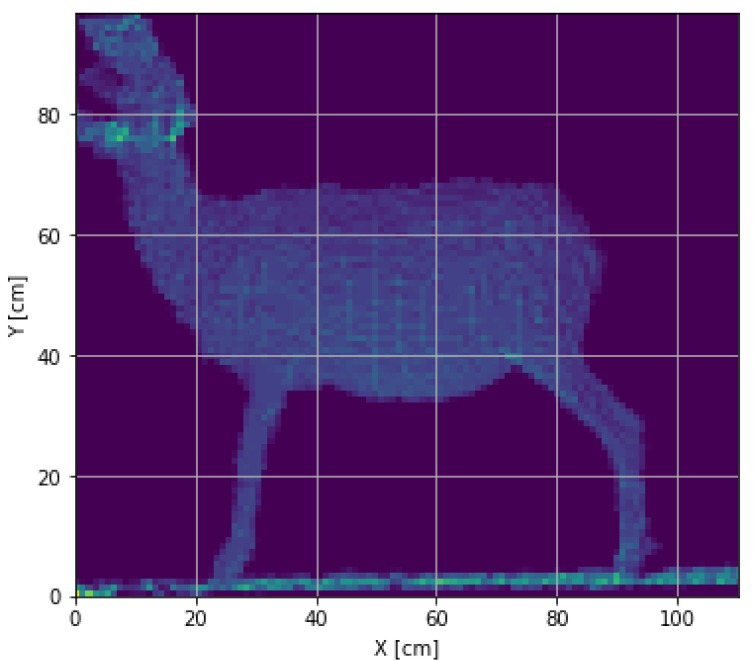
Segmented depth histogram on the Z-axis.

**Figure 7:**
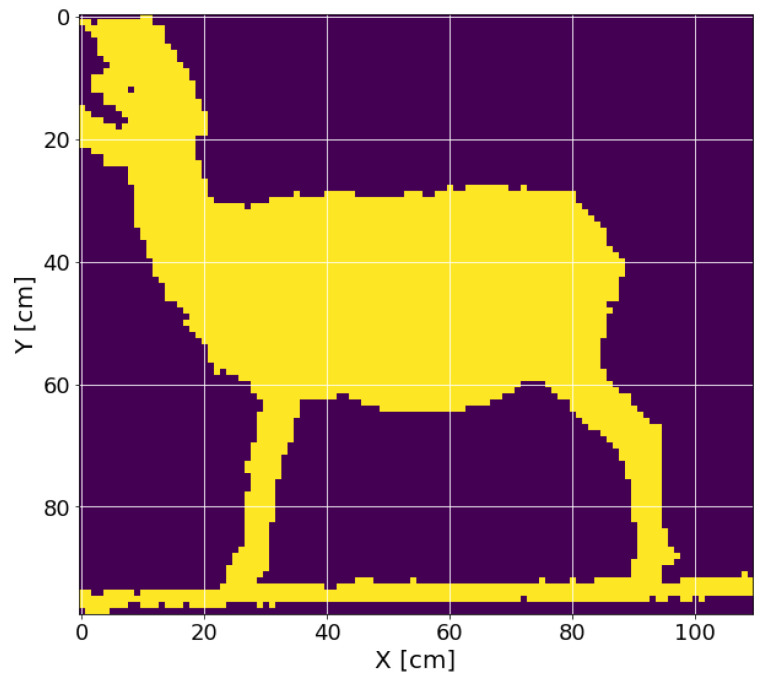
Two-dimensional image with the information on the X-axis and the Y-axis.

In order to have only the sheep’s information and eliminate the background, a threshold cut was made on the Z-axis, removing the depth information of less than 1.2 and beyond 1.8 m. The appropriate value of the threshold was through multiple tests with the expected measurement conditions, and a two-dimensional histogram was obtained (Figure 6); this histogram was then converted to a 2D image with the conversion of 1cm equivalent to 1 pixel (Figure 7).

**Figure 8:**
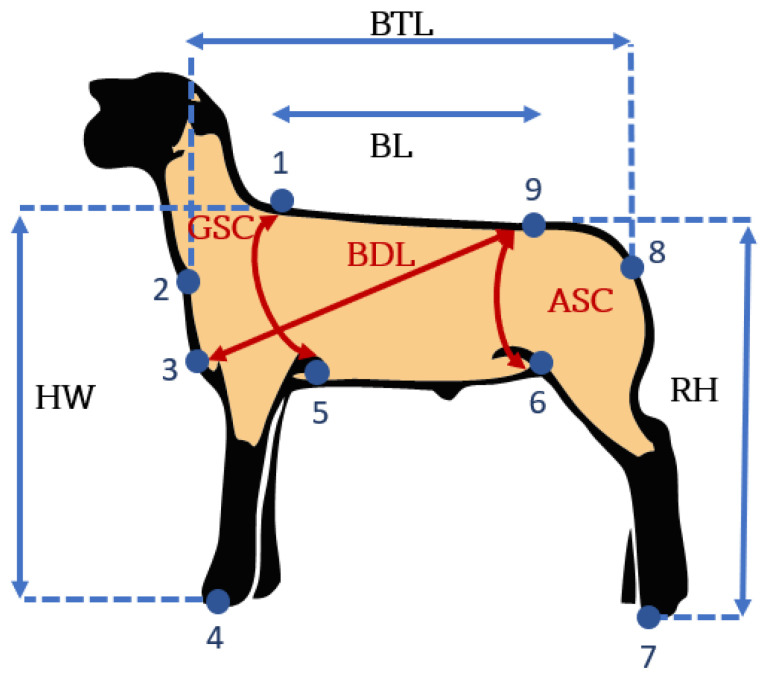
Schematic of the body measurements.

**Figure 9:**
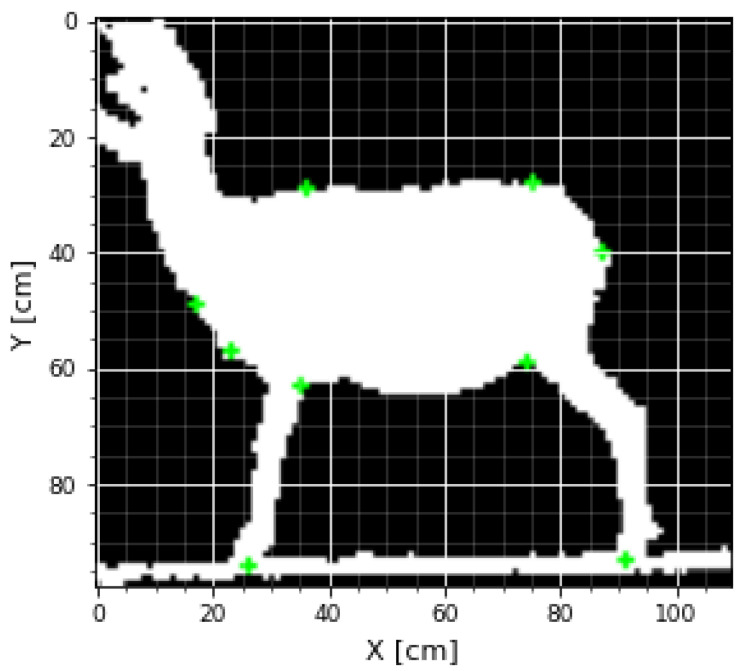
Interface where the user places the points of interest to generate the measurements.

Figure 8 shows the points and distances of interest to make the measurements. These points were selected from Figure 7, which was calibrated in centimeters. The points of interest were extracted with OpenCV tools showing the image, and the user only has to place a small cross on the points of interest measurements, which were stored automatically (Figure 9).

**Figure 10:**
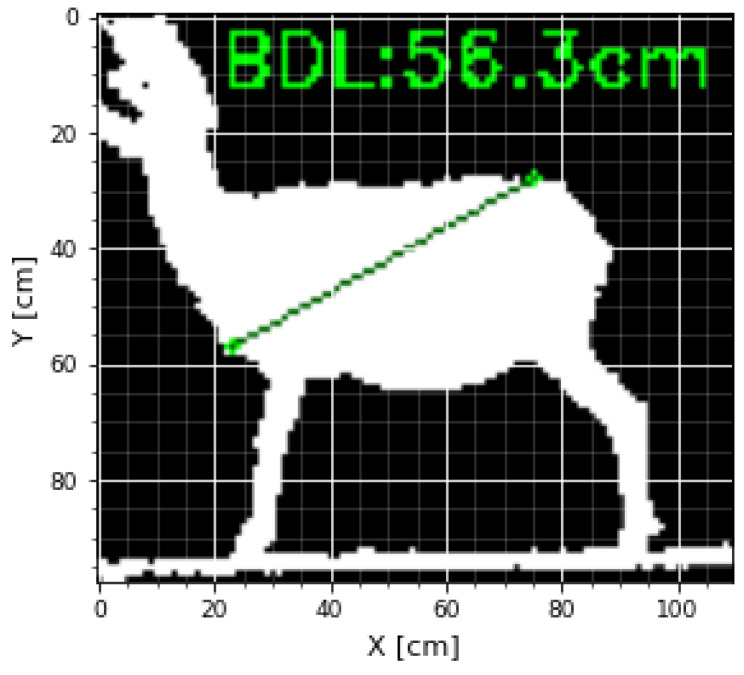
Example of BDL obtained by the Kinect^®^ sensor.

Then, these measurements were taken, and the calculations were executed considering that the scale for both the X- and Y-axes was in centimeters, that is 1 pixel is equal to 1cm; the Euclidean distances for each pair of points were previously defined; an example can be seen in Figure 10.

### 2.3. Body Measurements

The following Body Measurements (BMs) were recorded after data acquisition with the Kinect^®^ sensor for each animal. The BMs were taken as described previously by [59]: Height at Withers (HW), Body Length (BL), Diagonal Body Length (BDL), Total Body Length (BTL), Rump Height (RH), Abdomen Semi-Circumference (ASC), Girth Semi-Circumference (GSC) (Figure 8). The letter K was added at the end to differentiate the measurements obtained by the Kinect^®^ sensor. For example, the Height at Withers obtained by the Kinect^®^ would be HWK.

All BMs were recorded in cm. A flexible fiberglass tape was used for the measurements. For BW, the animals were weighed using a digital scale (EQB Model, Torrey^®^, Monterrey, Mexico).

### 2.4. Polynomial Model for Weight Estimation

In the current study, a polynomial model is proposed. This type of model fits the data to a non-linear model. The biggest problem is the adjustment of the coefficients and the proper determination of the grade. This research used a polynomial model based on the biometric variables of the sheep to estimate the weight. The expression proposed with these variables is shown in Equation (1), and the variables and their nomenclature are shown in Table 1.

(1) $$BWE=a_{1}HWK^{a_{8}}+a_{2}RHK^{a_{9}}+a_{3}BLK^{a_{10}}+a_{4}BDLK^{a_{11}}+a_{5}BTLK^{a_{12}}+a_{6}GSCK^{a_{13}}+a_{7}ASCK^{a_{14}}$$

The objective was to find the coefficients and exponents $a_{n}$ that minimize the actual and estimated weights’ error. The Root-Mean-Squared Error (RMSE) was used as a performance measure, defined by Equation (2). The solutions with a lower RMSE have a higher probability of hatching. A solution with an RMSE of 0 implies a prediction equal to the actual value. The RMSE magnifies errors of greater magnitude, but reduces those of lower magnitude. The errors expected in the estimates and experimental measurements are errors of low magnitude. Thus, the RMSE is an appropriate indicator for evaluating the algorithm’s performance.

(2) $$RMSE=\sqrt{\frac{\sum_{i=1}^{n}{\left(BWE-BW\right)}^{2}}{n}}$$

Metaheuristic search algorithms were used to estimate the 14 unknown coefficients. A significant advantage of using this type of algorithm is that it leaves the polynomial degree as an unknown. Given the complexity of the model, a logical option for flexibility and adaptability is metaheuristic algorithms. In this way, the metaheuristic algorithms analyze in each iteration the performance of the different degrees in each variable. For both algorithms, the same search limits were used. In the case of the coefficients, the search was limited between 0 and 5 and the coefficient values between 0 and 1.

### 2.5. Genetic Algorithm

One of the two algorithms used was a Genetic Algorithm (GA). This type of algorithm is inspired by the selection genes in natural selection. Usually, the individuals with the best genes have the most offspring. Under this principle, the search for the coefficients is carried out, with the best genes representing the combinations of coefficients and exponents that generate a lower RMSE.

This algorithm was chosen because it is the most extensive and widely used metaheuristic algorithm despite being a first-generation algorithm. Although there are multiple adaptations for its improvement, it is well known that all metaheuristic algorithms risk falling into local optima. This situation happens because they find a suitable solution and keep searching around that solution in the search space, but better solutions exist elsewhere in that space. The genetic algorithm is particularly prone to this because several specific search parameters must be tuned correctly, such as mutation percentage and individuals that can reproduce.

The version of this algorithm used is shown as pseudo-code in Algorithm 1. It goes through a stage of selection, one of mutation, and one of elitism. The complete search parameters used are shown in Table 2. These parameters were obtained through multiple runs based on the performance of the algorithm; however, it is important to note that there are various tuning methods such as CRS-Tuning [60], F-Race [61,62], and REVAC [63], which can be used to optimize the algorithm and other optimization techniques [64]. In particular, the use of these methods with a larger sample is recommended to compare the error reduction in future works.
**Algorithm 1** Genetic algorithm with elitism1:**Begin**2:Generate initial population P based on random vectors with coefficients and exponents3:**while** fitness evaluations consumed limit <40,000 **do**4:    Evaluate fitness of P members5:    Selection of best P members based on their fitness6:    Crossover of best parents from P based on a random single point7:    Mutation in a part of the individuals based on a probability value8:    Create new population P based on most-fit individuals and elitism9:**end while**10:Save the best vector11:**end**

### 2.6. Cuckoo Search Algorithm

The Cuckoo Search Algorithm (CSA) is based on how cuckoo birds reproduce. For this, it uses other birds’ nests and places its eggs in them, hoping that the other birds will raise their young. Of course, some eggs will be discovered, but the more the foreign eggs resemble the host bird’s eggs, the more likely they will hatch. This behavior has been successfully adapted to a metaheuristic algorithm, an egg being in a nest being a possible solution.

The CSA has been widely used and has shown significant improvement combined with the Lévy flight. In this work, we decided to use this algorithm to verify that the hyperparameters used in the genetic algorithm did not affect the performance of the final model. The advantage of the CSA is that it contains only one specific parameter, which is the probability that the impostor egg will be discovered.

It should also be considered that it was not the objective of this study to demonstrate the superiority of one algorithm over another. In fact, according to the no free lunch theorem, all optimization algorithms have the same average performance [65]. This supposition implies that if an algorithm was more efficient in one task, it will be worse for another task. Therefore, tests must be carried out with at least two algorithms, for which the CSA was chosen to compare the results with the GA. The complete process of the algorithm is shown in the pseudo-code in Algorithm 2, and the search parameters are shown in Table 3.
**Algorithm 2** Cuckoo search algorithm1:**Begin**2:Create random initial population of n host nests (vectors with coefficients and exponents)3:**while** fitness evaluations consumed limit <40,000 **do**4:    Obtain a cuckoo randomly by Lévy flights and evaluate fitness $F_{i}$5:    Choose a nest among n (say, j) randomly6:    **if** $F_{i}$>$F_{j}$ **then**7:        Replace j with the new solution8:    **end if**9:    Abandon a fraction $P_{a}$ of the worse nests and build new ones at the new values via Lévy flights10:    Rank the solutions and find the current best11:**end while**12:Save the best vector13:**end**

The same general parameters and same fitness evaluations consumed were used to make a fairer comparison between the algorithms [66,67,68].

## 3. Results

**Figure 11:**
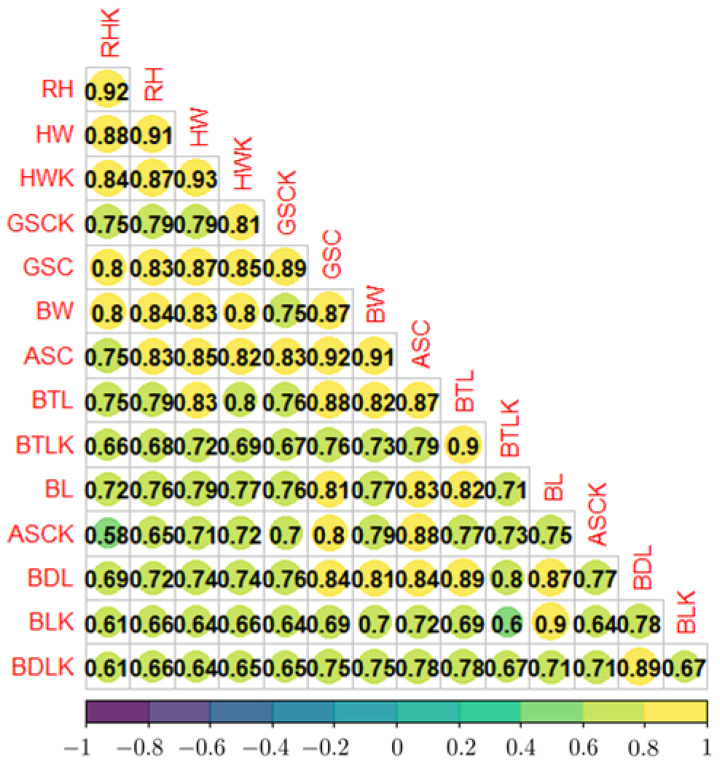
Correlation matrix of the biometric parameters.

To understand the proximity of the measurements obtained with the Kinect^®^ sensor and by manual methods, the calculation of the Pearson correlation coefficients was performed. Figure 11 shows the results of this correlation test.

Examining the pairs of values obtained, between the Kinect^®^ and the corresponding ones by the manual method, they were found to range from 0.83 to 0.93, for HW and ASC, respectively, having an average of 0.90.

The two algorithms were executed with the parameters of Table 2 and Table 3. The results of the coefficients and exponents for both algorithms are presented in Table 4.

This implies that the model obtained by the genetic algorithm is described by Equation (3), while the CSA is represented by Equation (4).

(3) $$\begin{array}{cc} 0.057{\left(HWK\right)}^{4}+0.209{\left(RHK\right)}^{3} & +0.22{\left(BLK\right)}^{4}+0.009\left(BDLK\right)\\ & +0.548{\left(BTLK\right)}^{2}+6.4\times^{-4}{\left(GSCK\right)}^{4}+0.026{\left(ASCK\right)}^{4} \end{array}$$

(4) $$\begin{array}{cc} 0.198{\left(HWK\right)}^{4}+0.198{\left(RHK\right)}^{2} & +0.023{\left(BLK\right)}^{5}+0.189{\left(BDLK\right)}^{5}\\ & +0.422{\left(BTLK\right)}^{2}+0.035{\left(GSCK\right)}^{4}+0.0419{\left(ASCK\right)}^{3} \end{array}$$

With the previous equations, the statistical errors (Table 5) were obtained.

A cross-validation stage was required since the methods are based on random values. Therefore, 10 runs were carried out, and the average statistical values of these runs are shown in Table 6.

**Figure 12:**
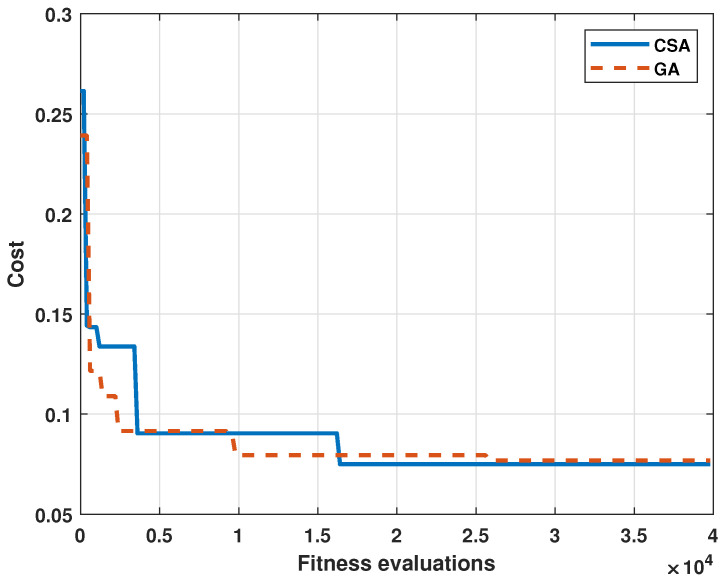
Function costs for both algorithms.

The convergence curves obtained for both algorithms are shown in Figure 12.

**Figure 13:**
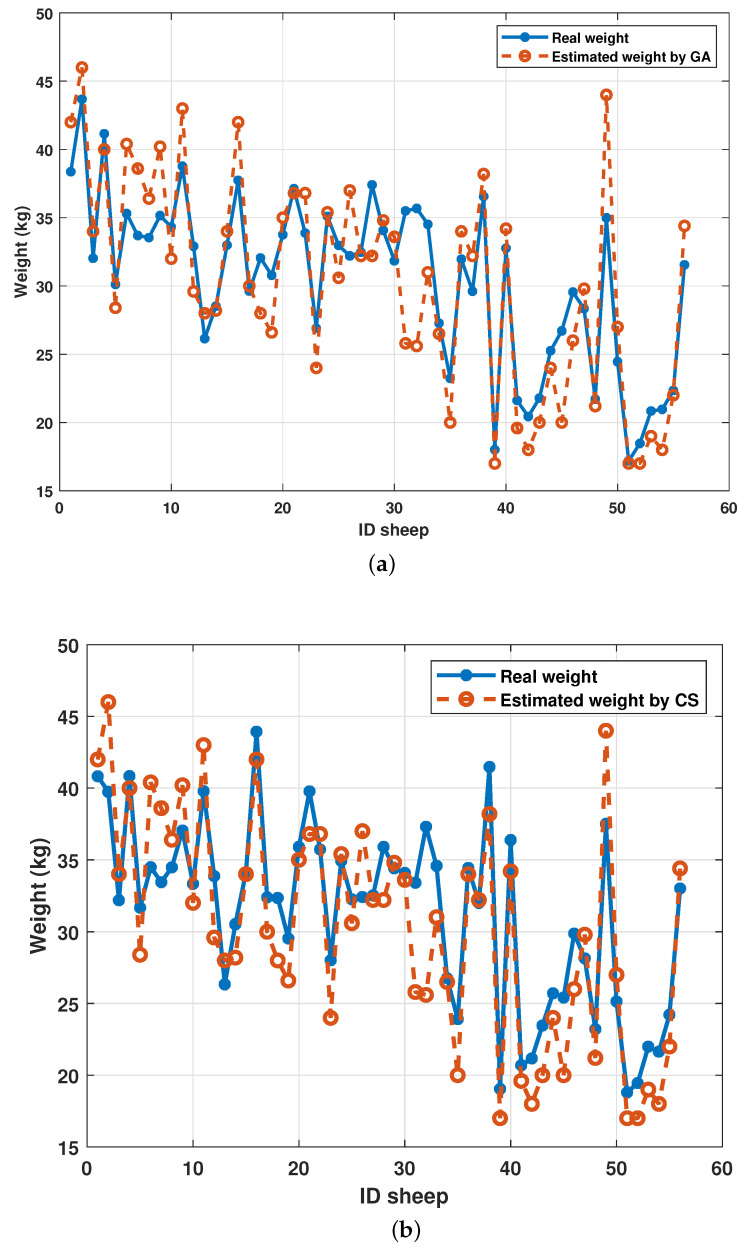
Comparison between real weights’ values and estimated weights. (**a**) Comparison using the genetic algorithm; (**b**) Comparison using the cuckoo search algorithm.

Finally, the comparison of the error between the real values and the estimated ones is given in Figure 13.

The results are discussed in depth in Section 4.

## 4. Discussion

Numerous studies show that biometric measurements are highly relevant for predicting the body weight of sheep, and many of them use different techniques such as Multiple Linear Regression (MLR), which is very simple and the most used, and in recent years, machine learning techniques. Nevertheless, there is a lack of studies in the literature using metaheuristic algorithms, but it is possible to compare the predictive accuracy of our approach based on the $R^{2}\left(\%\right)$ values. In the case of models based on machine learning, Huma and Iqbal [69] proposed using seven biometric parameters for different models: regression trees (85.4%), linear model (86.1%), support vector machine (89.7%), and random forest (91.6%); it is necessary to mention that they used 131 Balochi sheep from Pakistan, and all of them were males, so all the values obtained were higher than those obtained in our study: 79.23% and 79.98% (Table 5). Canul-Solis et al. [70] used only one parameter to estimate the BW; this was the hip width, and they proposed three equations: linear, quadratic, and exponential, obtaining 96%, 96%, and 95%, respectively; in this study, 577 Pelibuey ewe lambs were used. Ansar et al. [71] estimated an $R^{2}\left(\%\right)$ value of 64.5% for the Chi-squared Automatic Interaction Detector algorithm (CHAID) in the BW prediction based on nine parameters; for this study, they used Thalli sheep, while Mohammad et al. [72] estimated a higher $R^{2}\left(\%\right)$ value of 72% for the CHAID algorithm, based on four parameters; in this case, indigenous sheep breeds from Pakistan were used. Roel et al. [73] carried out a study that used images of heifers, and they were processed using computer vision techniques to predict weight using deep neural networks; they obtained two models depending on the view: the side-view model had a coefficient of determination $R^{2}\left(\%\right)$ of 91%, and the top-view model had an $R^{2}$ of 0.96%. Samperio et al. [74] proposed a system for estimating weight by means of 3D zenithal images from 272 lambs, obtaining an $R^{2}\left(\%\right)$ of 86%. Other studies used the Kinect^®^ sensor to estimate the BW, as was the case of Pezzuolo et al. [75]; this study proposed the use of two Kinect^®^ sensors to estimate the BW of pigs, one for the lateral view and the other for the top section, where they obtained an $R^{2}\left(\%\right)$ of 95%. Our proposal has the advantage of using a single Kinect^®^, which simplifies the acquisition of the images. Finally, Reference [32] is a review of studies based on CV that were carried out using algorithms based on linear regression and artificial neural networks for weight estimation. However, those studies did not mention the metaheuristic algorithms used in this work.

As shown in Table 7, previous works showed simple empirical formulas or models that cannot take advantage of all the variables that can be measured simultaneously with artificial vision.

The results in the measurement of the parameters via CV can be seen in the correlation matrix of Figure 11. It can be seen that the biometric parameter with the best correlation to the BW was RHK with 0.80. With all the parameters in this range [0.7 to 0.8], each biometric parameter used correlated highly with the BW. The real parameters and those obtained with the Kinect^®^ sensor showed a minimum value of 0.83 and a maximum value of 0.93 for ASC and HW, respectively. These values might indicate that the measured values with respect to the real value have an average error of less than 10%. However, there were some atypical observations due to disturbances, such as sudden movements of the animals during the measurements. This situation caused some values, such as ASC in Sheep 31 and 32, to have atypical measurement errors: 50% and 52%, respectively. This error was reflected in the weight prediction by the algorithm, obtaining errors for those points of 25.7% and 27.7%. These point errors for Sheep 31 and 32 were still lower than the average reported, for example, by the Schaefferformula for Jersey Cross cattle body weight (kg), which was around 31%.

Table 5 shows the statistical indicators obtained by each algorithm. The performance of each algorithm was measured with the RMSE, defined by Equation (2). The GA obtained an RMSE of 7.68%, and the CSA obtained an RMSE of 7.55%. The difference between both algorithms was 0.13%. Regarding the correlation coefficient $R^{2}$, the value for GA was 79.23% and 79.98%. This is a difference of only 0.75%. Although each algorithm found polynomials with a similar performance reflected in the RMSE and $R^{2}$, each algorithm found a different local optimum. The objective of using two different metaheuristic algorithms was to compare other solutions with similar performance. Additionally, it was observed that, at the end of the process, neither algorithm presented a reduction of the RMSE. Thus, it is suggested for future or similar works to reduce the number of fitness evaluations.

The previous point was demonstrated by the results of both algorithms since the local optimum found by each one reflected a similar performance; however, it is observed in Table 4 that the parameters of each polynomial were different. Figure 12 demonstrates the performance of each algorithm in the search for the solution with the best fitness. While the GA converged to its optimum in fewer fitness evaluations, the CSA had a slightly better optimum. It can be summarized that the local optima for both algorithms were similar in performance.

Finally, Figure 13 shows the performance as a weight estimator of each algorithm. Both algorithms presented similar prediction trends. The sheep with ID 31 and 32 presented the largest errors in both algorithms. If they were considered atypical points, the accuracy of the algorithms would increase.

## 5. Conclusions

This research developed an intelligent estimator system based on evolutionary computation. A multivariate polynomial with an adjustable degree was adjusted based on the biometric parameters of Pelibuey sheep using metaheuristic algorithms. The polynomial models presented an RMSE of 7.68% for the GA and 7.55% for the CSA.

Methods based on CV to obtain biometric measurements were carried out without performing manual measurements. The approach showed an average of 0.9 in the measurements obtained from the Pearson correlation coefficient.

The results are encouraging, as they presented an estimate with an RMSE of less than 8% for both cases. Instead of using artificial intelligence techniques such as ANNs, the presented methodology is an alternative approach to estimating Body Weight (BW). A more significant amount of data is required and the errors are expected to be reduced with a more extensive database used to fit the multivariate polynomial. However, this type of study serves as a background to carry out more extensive investigations. On the other hand, this research can be improved with the use of hardware with higher performance. Another point implies obtaining a more extensive image preprocessing stage and a fixed data acquisition system, where the biometric parameters of a large number of sheep can be taken fluidly.

The proposed computational model was supported by artificial intelligence techniques, which can be difficult to adapt in microsystems. However, the proposed model is a polynomial, which is easily processed by any embedded device. On the other hand, OpenCV and the biometric parameters option can also be used for implementation in compatible microsystems in Python.

## Figures and Tables

**Table 1:** Biometric variables used as elements of the polynomial model.

Biometric Variables	Nomenclature
Height at Withers sensed by Kinect^®^	HWK
Rump Height sensed by Kinect^®^	RHK
Body Length sensed by Kinect^®^	BLK
Body Diagonal Length sensed by Kinect^®^	BDLK
Body Total Length sensed by Kinect^®^	BTLK
Girth Semi-Circumference sensed by Kinect^®^	GSCK
Abdomen Semi-Circumference sensed by Kinect^®^	ASCK
Body Weight Estimated	BWE

**Table 2:** Search parameters used for the genetic algorithm.

Parameter	Value	Description
Population size	200	Number of individuals (random solutions)
Termination condition: fitness evaluations consumed limit	≤40,000	Maximum number of times the fitness function is evaluated
Biological pressure	85%	Percentage of individuals that reproduce
Elitism	10%	Percentage of best individuals whose reproduction is guaranteed
Mutation probability	30%	Probability that an individual will mutate
Crossover type	Single-point crossover	A random point in a parent is selected to combine the two parents
Selection method	Rank selection	The individual with the best performance has the best rank; the individual with the best rank has a higher probability of reproducing
Coefficient search range	[0–1]	Float limits values for each coefficient
Exponent search range	[0–5]	Integer limits values for each exponent

**Table 3:** Search parameters used for the cuckoo search algorithm.

Parameter	Value	Description
Termination condition: fitness evaluations consumed limit	≤40,000	Number of times the fitness function is evaluated
Nests	200	Population size
Eggs	$a_{n}$	Random coefficients and exponents
Pa	25%	Probability of foreign eggs being discovered
Coefficient search range	[0–1]	Float limits values for each coefficient
Exponent search range	[0–5]	Integer limits values for each exponent

**Table 4:** Results of the coefficients and exponents obtained for both algorithms.

Coefficients	GA	CSA
$a_{1}$	0.057	0.198
$a_{2}$	0.209	0.198
$a_{3}$	0.220	0.023
$a_{4}$	0.009	0.189
$a_{5}$	0.598	0.422
$a_{6}$	6.4 × 10${}^{-4}$	0.035
$a_{7}$	0.026	0.0419
$a_{8}$	4	4
$a_{9}$	3	2
$a_{10}$	4	5
$a_{11}$	1	5
$a_{12}$	2	2
$a_{13}$	4	4
$a_{14}$	4	3

**Table 5:** Statistical errors of the models obtained by the GA and CSA.

Estimator	GA	CSA
RMSE (%)	7.68	7.55
$R^{2}$ (%)	79.23	79.98
MBE (%)	0.33	2.26
MAPE (%)	9.45	9.97

**Table 6:** Cross-validation with 10 runs for the GA and CSA.

Estimator	GA	CSA
RMSE (%)	8.83	9.68
$R^{2}$ (%)	72.46	66.59
MBE (%)	1.43	0.95
MAPE (%)	10.83	11.2

**Table 7:** Mathematical models.

Technique	Equation	References
Agarwal’s formula ^a^	W=(Girth×length)/Y	[76]
Schaeffer’s formula ^b^	$W=(L\times G^{2})/300$	[76]
Logistic model ^c^	$Y=A{\left(1+e^{-Kt}\right)}^{-M}$	[77]
Gompertz ^c^	$Y=Aexp(-Be^{-kt})$	[78]
Von Bertalanffy ^c^	$Y=A{\left(1-Be^{-kt}\right)}^{3}$	[77]

^a^*Y* is equal to 9.0 if the girth is less than 65 inches; *Y* is equal to 8.5 if the girth is between 65 and 80 inches; *Y* is equal to 8.0 if the girth is over 80 inches. ^b^ *L* is the length of the animal from the point of the shoulder to the pin bone in inches, and *G* is the chest girth of the animal in inches. ^c^ *A* is the body weight (asymptotic), namely the value of t approaches infinity; *B* is the scale parameter (the value of the integral constant); *e* is the logarithm base (2.718282); *k* is the average rate of growth of the body until the animal reaches body maturity; *M* is the value of the function in the search for the inflection point (curve shape); *t* is the time in units of the month.

## Data Availability

The data presented in this study are available upon request from the corresponding author.

## Abbreviations

The following abbreviations are used in this manuscript:

BW	Body Weight
MA	Metaheuristic Algorithms
CV	Computer Vision
RMSE	Root-Mean-Squared Error
GA	Genetic Algorithm
CSA	Cuckoo Search Algorithm
DM	Dry Matter
HW	Height at Withers
HWK	Height at Withers sensed by Kinect^®^
BL	Body Length
BLK	Body Length sensed by Kinect^®^
BDL	Body Diagonal Length
BDLK	Body Diagonal Length sensed by Kinect^®^
BTL	Body Total Length
BTLK	Body Total Length sensed by Kinect^®^
RH	Rump Height
RHK	Rump Height sensed by Kinect^®^
ASC	Abdomen Semi-Circumference
ASCK	Abdomen Semi-Circumference sensed by Kinect^®^
GSC	Girth Semi-Circumference
GSCK	Girth Semi-Circumference sensed by Kinect^®^
ANN	Artificial Neural Network
